# The National Dental Practice-Based Research Network Dental Implant Restoration Registry to Evaluate Dental Implant Outcomes in Community Practice Settings: Protocol for a Prospective Observational Study

**DOI:** 10.2196/82795

**Published:** 2026-02-27

**Authors:** Joana Cunha-Cruz, Danyelle Barton, David Cochran, Phillip Crawford, Farahnaz Fahimipour, Ellen Funkhouser, Nicolaas C Geurs, Gregg H Gilbert, James D Johnson, Ning Smith, Kaur Maninder, Sarah Jane Startley, Kimberly Stewart, Lisa Waiwaiole, National Dental PBRN Collaborative Group, Muna Anabtawi

**Affiliations:** 1 Department of Clinical and Community Sciences, School of Dentistry, University of Alabama at Birmingham, 1717 11th Avenue S, Suite 402E, Birmingham, AL, United States, 1 205-996-5298; 2 Kaiser Permanente, Portland, OR, United States; 3 Department of Periodontology, University of Texas Health San Antonio, San Antonio, TX, United States; 4 Department of Periodontology, School of Dentistry, University of Alabama at Birmingham, Birmingham, AL, United States; 5 College of Dentistry, University of Florida, Gainesville, FL, United States; 6 See Acknowledgments

**Keywords:** dental implants, dental prosthesis, dentures, implant supported, oral health, prospective studies, practice-based research

## Abstract

**Background:**

Dental implants are a widely used therapeutic option for tooth replacement; however, biological and prosthetic complications may compromise implant success. While prior research has largely focused on academic or specialty settings, data on implant outcomes in community dental practices remain limited.

**Objective:**

This study aims to establish a national registry within the National Dental Practice-Based Research Network to evaluate the incidence of and factors associated with biological and prosthetic complications following implant therapy in community practice settings.

**Methods:**

This prospective observational cohort study plans to include approximately 2000 implant restorations from 1550 patients receiving implant restorations from 150 practitioners across 6 regional network nodes. Clinical, implant, and prosthetic characteristics will be recorded at baseline, with annual follow-up visits collecting data on complications, implant failures, prosthetic issues, and patient-reported outcomes. Digitized radiographs will be centrally reviewed for peri-implant bone changes, emergence angle, and prosthetic fit. The primary outcome is the incidence of biological and prosthetic complications. Secondary analyses will evaluate patient-centered outcomes and identify risk factors for complications. Multilevel mixed-effects regression and time-to-event analyses will be used to account for clustering and censored data.

**Results:**

Of 430 practitioners who expressed initial interest, 187 (43.5%) completed training and began patient enrollment, with 143 (76.5%) of these practitioners enrolling at least one participant. A total of 1453 patients were registered between August 2022 and October 2024. Practitioner activity and enrollment peaked in late 2023. Overall, 1178 (81.1%) baseline patient surveys and 1453 (100%) baseline clinical forms were completed, covering 2087 dental implant restorations.

**Conclusions:**

The registry will generate robust, real-world evidence on implant-related complications and their predictors in community practice. The findings will enhance clinical decision-making; support personalized risk assessment; and, ultimately, improve patient outcomes in implant dentistry.

## Introduction

Dental implants have become a standard treatment modality for rehabilitating partially and fully edentulous patients worldwide. The global dental implant market, valued at approximately US $9 billion in 2025 with an estimated 5 million implants placed annually, is projected to experience steady growth due to rising patient demand and advances in implant technologies [1]. While dental implants have demonstrated long-term success in many cases, they are not free from complications associated with various factors, including treatment planning, patient-related issues, surgical and prosthetic execution, material failure, and lack of maintenance [2-5].

The longitudinal survival rates of osseointegrated dental implants range between 90% and 96.2% after 5 years [2-5]. However, these figures may not fully capture the rates of peri-implant disease and health. Biological and prosthetic complications can interfere with the health of the peri-implant tissues and the function and aesthetics of implant restorations [6]. Peri-implant diseases are classified into peri-implant mucositis (inflammation restricted to the peri-implant mucosa) and peri-implantitis (characterized by peri-implant bone loss). The prevalence of peri-implant mucositis and peri-implantitis varies widely, with reported ranges of 19% to 65% and 1% to 47%, respectively, and meta-analyses estimating weighted mean prevalences of 43% to 63% for mucositis and 22% to 25% for peri-implantitis [7-12]. Prosthetic complications, such as screw loosening, fractures, chipping, and decementation, pose significant clinical challenges in implant dentistry. Their prevalence ranges from 10% to 16% during midterm follow-up influenced by prosthetic design and materials [13-15].

The current body of literature on biological and prosthetic complications is limited by small studies primarily conducted in European academic and specialty settings [4,16,17]. Many of these studies have been retrospective and may not accurately represent community practice settings. The National Dental Practice-Based Research Network (PBRN) Dental Implant Restoration Registry aims to address these knowledge gaps by creating an implant registry to record implant and prosthetic procedures and characteristics and any complications in community practice settings. The primary objective of this study is to quantify the incidence of biological and prosthetic complications in dental implants over a 3-year period. Secondary objectives are to assess risk factors associated with implant complications, develop a risk assessment tool for implant complications, and evaluate patient-reported outcomes related to implant therapy.

## Methods

### Study Design and Setting

This is a prospective observational cohort study designed to evaluate outcomes of dental implant restorations over a 3-year follow-up period in community practice settings. It will be conducted across approximately 150 dental practices in the United States. Eligible practitioners who are members of the National Dental PBRN from all 6 regional nodes and provide dental implant restorative therapy will be invited to participate. The study protocol and data collection instruments have been reviewed by the network’s Practitioner Executive Committee. The funding agency and the institutional review board (IRB) also reviewed and approved the protocol (version 6; November 11, 2024). This study protocol is reported following the SPIRIT (Standard Protocol Items: Recommendations for Interventional Trials) [18] reporting guidelines for protocols and the STROBE (Strengthening the Reporting of Observational Studies in Epidemiology) reporting guidelines for observational studies [19].

The National Dental PBRN is a nationwide initiative funded by the National Institute of Dental and Craniofacial Research (NIDCR) that engages dental practitioners from a broad range of community settings across the United States in conducting clinical research relevant to routine dental care [20,21]. The network is structured into 6 regional nodes, each housed at distinct academic or research institutions. Each node is led by node directors and supported by node research coordinators, who serve as the primary liaisons between the network and participating dental practitioners. Node coordinators facilitate communication, recruitment, and coordination of study activities, fostering close collaboration with dental providers to ensure study feasibility and relevance.

An administrative and resource center (ARC) housed at the University of Alabama at Birmingham oversees centralized functions, including ethical review through the central IRB, administrative agreements, and management of payments to study participants and dental practitioners acting as individual investigators. The ARC leads national engagement efforts and communication and dissemination activities. The Practitioner Executive Committee, composed of dental practitioner members from the network, provides critical input on study concepts, protocol development, and implementation strategies. The National Coordinating Center based at Kaiser Permanente Center for Health Research manages data systems, quality control, and monitoring activities. Funding for the network is coordinated through distinct requests for applications issued by the National Institutes of Health (NIH) and NIDCR; separate collaborative agreements fund the ARC, the National Coordinating Center, and individual research studies. Program officers from the NIH and NIDCR work closely with the network leadership across all components to ensure alignment and successful execution. This organizational structure promotes close collaboration among dental practitioners, researchers, research coordinators, data managers, biostatisticians, and principal investigator teams to facilitate the successful completion of studies.

### Study Population

Participant inclusion criteria are patients with planned receipt of dental implant restorations from a participating practice who are older than 18 or 19 years (depending on the regional node) and are available for the duration of the study. Exclusion criteria include an inability to comply with study procedures or provide informed consent. Each enrolled patient can contribute more than one implant if multiple implants will be restored at the baseline visit.

### Recruitment and Retention

Practitioners will recruit and enroll their patients during the prosthetic phase of implant therapy after they complete study readiness and training activities. The network’s regional coordinators will assist practitioners in implementing the study protocol and monitoring recruitment progress. Strategies to enhance recruitment include regular communication with participating practitioners, provision of recruitment materials, offering continuing education credits for study participation, and periodic recruitment progress reports to motivate practitioners. Patient retention will be promoted through reminder emails, SMS text messages, or telephone calls for follow-up visits and surveys, and flexible scheduling of follow-up visits.

### Study Schedule of Assessments

The study timeline includes a baseline visit (prosthesis insertion) for enrollment, consent, and baseline data collection, followed by annual follow-up visits at 1, 2, and 3 years (±30 days and +150 days from the target date).

### Data Collection Procedures and Variables

#### Overview

All study data will be collected electronically using secure, web-based forms accessible via tablets, smartphones, or computers. Practitioners will enter clinical examination and procedural data through electronic case report forms (CRFs) at baseline and during each annual follow-up visit [22,23]. Radiographs taken as part of routine clinical care at baseline (prosthesis placement) and at each follow-up visit will be uploaded by practitioners to a secure centralized electronic repository. Separately, enrolled participants will complete electronic surveys following the baseline visit and at each annual follow-up interval.

#### Patient-Completed Surveys

Participants will provide self-reported demographic data, including age, sex, race, ethnicity, insurance type, educational level, household income, and residential environment. They will also report on general health status and behaviors, including medical conditions, allergies, medication use, and tobacco product use (cigarettes, cigars, smokeless tobacco, and electronic nicotine delivery systems). Oral health–related data will include measures of oral health–related quality of life (OHRQoL) and symptoms of dry mouth.

OHRQoL will be assessed using 10 items with responses on a 4-point Likert scale (“never,” “sometimes,” “always,” and “not sure”). Items will query functional limitations (eg, restrictions on eating and difficulty with chewing or speaking), pain or discomfort during eating or oral hygiene, medication use for dental pain, social impacts, and satisfaction with dental appearance and implant-supported prostheses drawing on domains used in established OHRQoL instruments [24,25]. Dry mouth symptoms will be assessed using 4 questions covering frequency of dry mouth diagnosis, difficulty swallowing, oral dryness during meals, and burning tongue sensations.

At each annual follow-up, participants will update their health status and medication and tobacco use and recomplete the OHRQoL questions.

#### Practitioner-Completed Clinical Research Forms

Practitioners will complete standardized electronic CRFs at baseline and each follow-up visit to capture comprehensive patient-, implant-, and prosthesis-level data:

- Patient-level clinical data—at baseline, practitioners will assess and record the participants’ overall oral health, detailed periodontal status (health, gingivitis, and periodontitis stage and progression) around natural teeth, the presence and extent of peri-implant disease in existing implants, routine dental maintenance history (frequency of maintenance visits in the prior 12 months), and parafunctional habits (eg, bruxism and occlusal guard use).
- Implant-level characteristics—for each study implant, practitioners will document the reason for tooth loss at the implant site; implant brand, length, and diameter; timing of placement in relation to extraction; results of bone and soft tissue grafting; and any early healing complications.
- Peri-implant mucosal characteristics—practitioners will document their assessments of peri-implant soft tissue (bleeding on probing, presence of purulence, deepest probing depth and location, mucosal recession and its extent, and width of keratinized mucosa), patient-reported pain at the implant site (with pain characteristics), and radiographic evaluation of bone levels.
- Prosthetic characteristics—data will be collected on prosthetic connection type, temporary prosthesis use and timing, final prosthesis type (fixed or removable), retention method, material, occlusal scheme, classification of opposing dentition, number of units and pontics, cantilever presence, abutment and cement types, seating and fit, and the presence and location of open contacts.

At each follow-up, CRFs will be used to record changes in periodontal and peri-implant status, occurrences of peri-implant disease or other complications, updates on implant survival (removal and reason), patient-reported pain, and radiographic bone loss. Practitioners will also document any biological or prosthetic complications (eg, screw loosening or fracture, prosthesis or abutment misfit, loss of retention, chipping or fracture of restorative materials, cement remnants, and adverse events), as well as management procedures and outcomes.

#### Radiographic Measurement for the Assessment of Bone Level and Prosthetic Fit

Radiographs obtained at baseline (prosthetic placement) and at annual follow-up visits (1, 2, and 3 years) will be used to quantify peri-implant bone levels and evaluate the fit of implant-supported prostheses. Calibrated evaluators will measure bone levels and prosthetic fit using the NIH ImageJ software [26] following a standardized protocol, with annotated images and measurements securely saved and uploaded to the study database.

Alveolar bone levels will be measured at the mesial and distal surfaces of each study implant as the linear distance from the implant platform to the most coronal bone-implant contact visible on the radiograph. As images will originate from different clinical systems with varying resolutions, measurements will be recorded in pixels initially and converted to millimeters using implant-specific geometry parameters (implant length or diameter) to standardize measurements across images.

Radiographs will also be examined for gaps at the implant-abutment and abutment-prosthesis interfaces, with fit discrepancies recorded as a dichotomous variable. The emergence angle will be measured mesially and distally using the implant platform as the vertex and lines drawn along the implant’s long axis and the gingival contour of the abutment or prosthesis to assess prosthetic design and potential biomechanical risk factors.

### Data Management and Monitoring

All data will be entered directly into the electronic data capture system, which includes built-in quality control checks. All radiographs will be anonymized and reviewed centrally for quality assurance. Two independent and calibrated evaluators blinded to clinical data and each other’s measurements will perform radiographic measurements. Discrepancies exceeding predefined thresholds will prompt involvement of a third adjudicator, who will conduct an independent assessment. All measurement data and marked images will be stored in a centralized repository.

Data will be encrypted for transmission to the coordinating center and stored on password-protected computers. Unique study codes will be used for participants to maintain confidentiality. The data management team will perform regular data quality checks and generate reports on missing data, inconsistencies, and protocol deviations. Research coordinators will support practitioners in resolving data inconsistencies and minimizing missing data whenever possible.

The study team will implement a clinical site monitoring plan to ensure the quality and integrity of the data collected. The monitoring strategy will include remote and/or on-site monitoring, regular quality control assessments, and ongoing monitoring of protocol adherence. These procedures will maintain high standards of data quality and ensure that study conduct is compliant with all applicable human subject research and regulatory requirements.

Although the study is observational, participant safety will remain a priority. Safety monitoring will focus on unanticipated problems involving risks to participants, including unanticipated problems that meet the definition of a serious adverse event. All reported events will be followed up on by the study team until resolution or stabilization. The principal investigator will be responsible for overall study oversight, including monitoring and assessment of adverse events and unanticipated problems and ensuring data integrity and adherence to the approved study protocol.

### Dissemination and Data Sharing

Results from this study will be disseminated through multiple channels, including publication in peer-reviewed scientific journals, presentation at national and international dental conferences, dissemination to network practitioners through newsletters and webinars, and public release of findings through press releases and the network website. Authorship will be determined according to the International Committee of Medical Journal Editors guidelines. The network will acknowledge the efforts of all participating practitioners by listing them as a group author in primary publications. Deidentified participant-level data and statistical code will be made available after the publication of primary study results in the PBRN’s website consistent with NIH policy [27].

### Statistical Considerations

#### Sample Size

Approximately 1550 patients will be enrolled to achieve a sample of 2000 implants. This sample size was determined based on anticipated complication rates of 10% to 15% over 3 years, the desire to detect differences in complication rates as small as 5% between groups, accounting for clustering of implant restorations within patients and practitioners, and allowing for up to 20% loss to follow-up. The sample size provides 80% power to detect clinically meaningful differences in complication rates between subgroups, with a 2-sided α of .05.

#### Statistical Analysis Plan

The study’s primary outcomes will be the incidence of biological and prosthetic complications occurring over the 3-year follow-up period. *Biological complications* will be defined according to the following criteria:

- Peri-implant mucositis—presence of bleeding on probing and/or purulent exudate around the implant without concomitant radiographic bone loss
- Peri-implantitis—radiographic peri-implant bone loss will be calculated by comparing the bone level at each annual follow-up to the baseline measurement, with bone loss defined as a negative change in millimeters
- Implant failure—documented implant loss or planned removal as reported by practitioners during follow-up

*Prosthetic complications* will include any event compromising the aesthetics or function of the implant restoration. These complications encompass screw loosening or fracture, structural fractures, resin or porcelain fractures, loss of retention, abutment or implant misfit, and other adverse prosthetic events captured via practitioner-completed data forms.

*Patient-centered outcomes* are critical components in evaluating the overall success of dental treatments. Secondary outcomes will include patient-reported measures of satisfaction with implant aesthetics and function as well as OHRQoL.

Descriptive statistics will be presented to summarize baseline characteristics and outcome variables. A study flow diagram will illustrate the number of participants at each stage of the study, including reasons for exclusion, loss to follow-up, and other participant dispositions following reporting guidelines [18,19]. Incidence and prevalence rates of biological and prosthetic complications will be estimated per implant and per year of follow-up. The primary analysis will use multilevel mixed-effects models to account for clustering of implant restorations within patients and practitioners. Time-to-event analyses will be conducted for complication-free survival. Multivariable regression models incorporating patient characteristics, implant-specific factors, prosthetic details, and behavioral variables will be estimated to identify predictors of biological and prosthetic complications. Subgroup analyses will explore effect modification by implant and prosthesis type and patient demographics. Missing data will be handled using multiple imputation techniques. Sensitivity analyses will be conducted to assess the impact of missing data and loss to follow-up on the study conclusions.

### Ethical Considerations

The study will be conducted in accordance with the International Council for Harmonisation of Technical Requirements for Pharmaceuticals for Human Use guidelines, the Code of Federal Regulations on the Protection of Human Subjects (title 45, part 46), and the NIDCR Clinical Terms of Award. IRB approval will be obtained from the National Dental PBRN Central IRB at the University of Alabama at Birmingham, with local context review by the local IRBs from each regional node. Any modifications to the protocol that may impact the conduct of the study or patient safety will be submitted for IRB approval before implementation. Protocol amendments will be communicated to all participating sites and investigators.

Informed consent will be obtained verbally at the prosthetic placement visit by trained personnel who have completed study protocol and human subject research training. The consent process will include a thorough explanation of the study procedures, risks, and benefits and will be documented in the electronic data capture system. Patients will have the opportunity to ask questions before providing consent. Participant confidentiality will be maintained through the use of unique study codes, data encryption, and password-protected storage. Access to identifiable information will be restricted to authorized study personnel on a need-to-know basis. The principal investigator and study statisticians will have access to the final dataset. Other investigators may request access to deidentified data after the completion of the primary analyses.

A check in the amount of USD $25 will be provided to participants for each survey answered for up to 4 surveys (US $100) per participant.

All investigators will disclose any potential conflicts of interest related to the study. These will be reviewed by the institutional conflict of interest committee and managed appropriately.

## Results

Of the 430 practitioners who initially expressed interest in the study, 194 (45.1%) completed human subjects research training and fulfilled other research and administrative requirements. Subsequently, 187 (96.4%) of these practitioners were trained in the study protocol and data management procedures and were cleared to begin patient enrollment. Of those 187 practitioners, 143 (76.5%) enrolled at least one patient.

Of these 143, practitioners were distributed across the 6 regions: Southwest (n=38, 26.6%), South Atlantic (n=31, 21.7%), Midwest (n=28, 19.6%), South Central (n=18, 12.6%), Northeast (n=18, 12.6%), and Western (n=10, 7%). Practice settings varied, with 63 (44.1%) in solo private practices, 40 (28%) in small group practices (2‐4 dentists), 5 (3.5%) in large group practices (5 or more dentists), 18 (12.6%) in academic settings, and 17 (11.9%) distributed among managed care or corporate practices and community health centers. More than half (n=85, 59.4%) of practitioners had advanced or specialty training, including 51 (35.7%) who completed advanced general dentistry programs (such as an advanced education in general dentistry program or general practice residency), 32 (22.4%) holding fellowships or masterships with the Academy of General Dentistry, 13 (9.1%) in prosthodontics, and 35 (24.5%) in other specialties (eg, dental implantology, orthodontics, and dental public health). Most practitioners (n=131, 91.6%) identified themselves as general dentists providing primary care, whereas 12 (8.4%) identified themselves as specialists.

**Figure 1.**
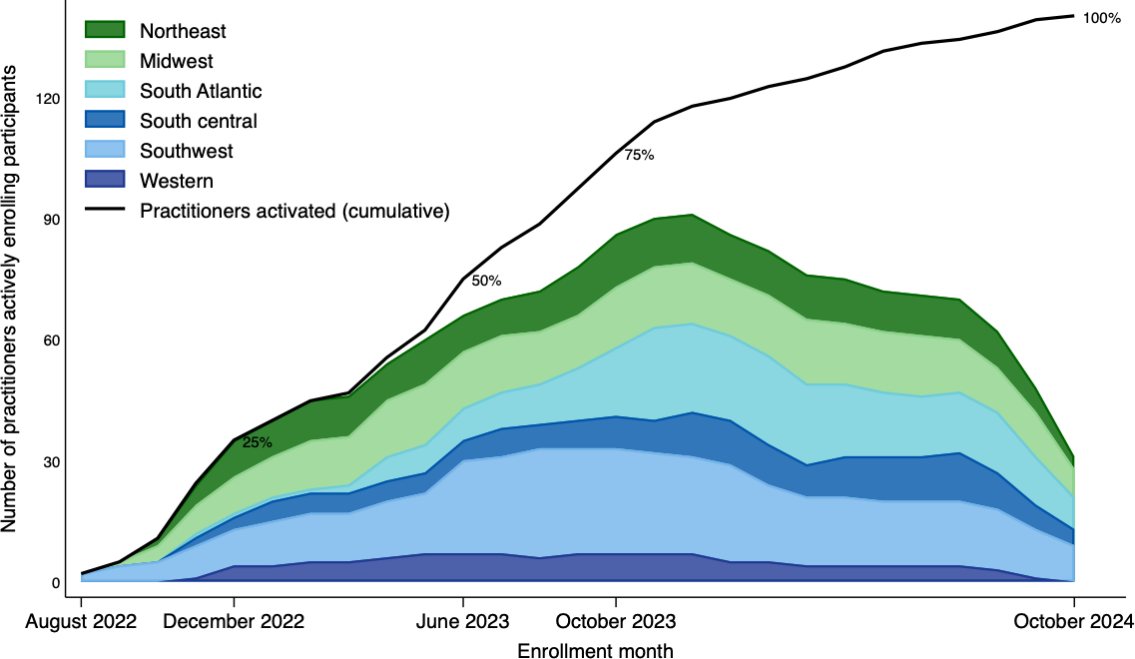
Percentage of active practitioners and monthly number of practitioners actively enrolling participants stratified by National Dental Practice-Based Research Network regional nodes: Dental Implant Restoration Registry from August 2022 to October 2024.

Practitioner activation and monthly enrollment activity increased rapidly after study launch in June 2022, with the number of practitioners actively enrolling participants increasing from 7 in August 2022 to more than 60 by mid-2023 and peaking at just under 90 in late 2023 (Figure 1). Monthly activity, assessed via the number of practitioners enrolling at least one participant, peaked between months 15 and 16, with 90 practitioners actively enrolling, and then declined to 31 by the final study month. The cumulative activation curve crossed the 25% and 50% thresholds by approximately December 2022 and June 2023, respectively, and surpassed 75% by the third quarter of 2023 as additional practices from all regions began enrolling; after the peak, the number of active enrolling practitioners declined gradually until October 2024 even as the cumulative number of active practitioners continued to increase to 100%.

**Figure 2.**
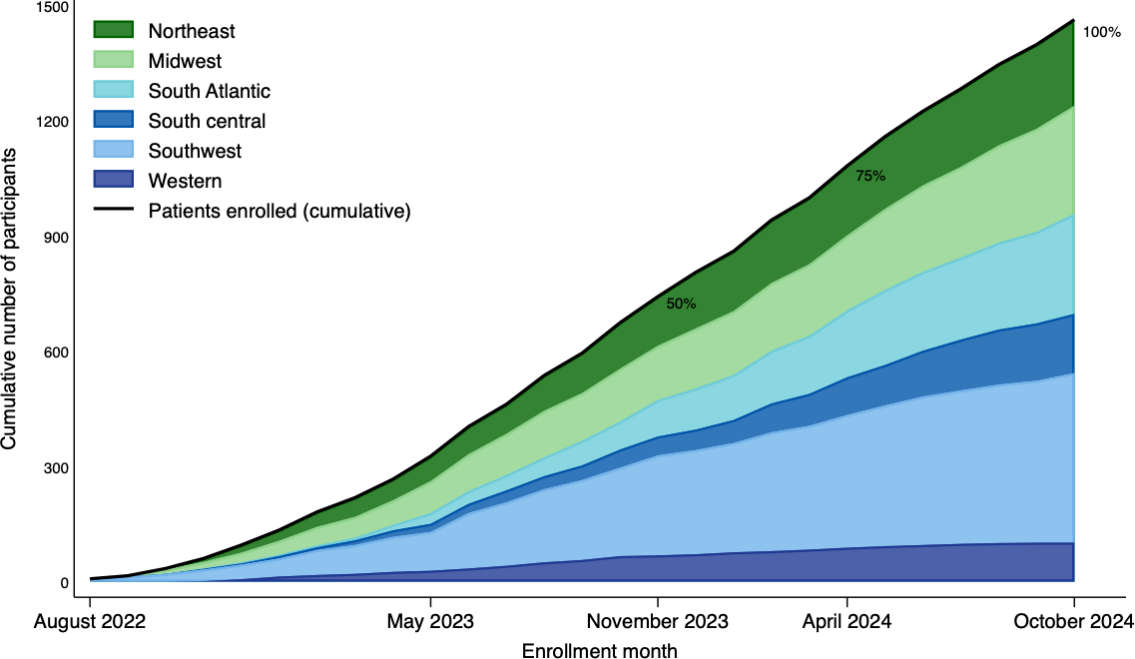
Number of participants enrolled in the Dental Implant Restoration Registry stratified by National Dental Practice-Based Research Network regional nodes.

Cumulative patient enrollment began gradually and increased steadily across all 6 regional nodes. The southwest and midwest nodes contributed the largest shares, producing a near-linear rise in total accrual over time. The cumulative enrollment curve crossed 25% of the final sample by June 2023 (month 11), 50% by November 2023 (month 15), and 75% by April 2024 (month 21), reaching the target sample by October 2024 (Figure 2).

On average, each practitioner enrolled 10 (SD 8.5) patient participants. The median number per practitioner was 8 (IQR 4-13), with a range from 1 to 39 participants. On average, each practitioner enrolled 14.6 (SD 12) implant restorations, with a median of 11 (IQR 5-21). The maximum number of implant restorations enrolled by a practitioner was 54.

The total number of participants enrolled in the registry was 1453. Practitioners completed all baseline CRFs for 2087 dental implant restorations, and 1178 (81.1%) participants completed their baseline surveys.

## Discussion

This paper details the successful establishment and operational metrics of a large-scale, geographically diverse, prospective dental implant registry in the United States. Over a 26-month period (August 2022 to October 2024), the registry enrolled 1453 participants and more than 2000 dental implant restorations. This achievement was driven predominantly by general dentists operating in solo and small group private practices, reflecting the real-world clinical context in the United States. Practitioner activation and patient enrollment peaked later in the study, with the 75% patient milestone reached in month 21. Methodological rigor was maintained, with completion of all baseline CRFs by practitioners and an 81.1% (1178/1453) data completion rate for patient surveys.

A key methodological weakness in prior studies of dental implant outcomes is their reliance on data from university and specialized implant clinics, which limits generalizability to general practice settings [2,7]. This registry addresses this limitation by engaging practitioners from diverse community settings across 6 US regions, positioning the cohort as representative of real-world effectiveness in implant therapy [8,9]. The final sample size is considerably larger than those of many previous epidemiological studies in implantology, which have typically included between 100 and 662 patients [7,8]. This scale overcomes the issue of limited sample size often cited in the literature [7] and provides a robust foundation for assessing outcomes and validating risk factors for peri-implant diseases [9].

The recruitment dynamics observed—a phased approach over 26 months with a late peak in activity—mirror the operational challenges typical of pragmatic trials [28]. The need to enroll and sustain the engagement of numerous practicing clinicians often leads to initial enthusiasm waning, resulting in premature discontinuation due to poor recruitment [29]. This registry overcame these challenges through staggered training, ramp-up periods, and ongoing activation of new practitioners throughout the enrollment period. This approach aligns with the operational model of the PBRN [21]. The rapid increase in activity after the initial launch phase demonstrates the effectiveness of the training and support infrastructure provided to participating sites.

The enrollment window, with key accrual milestones reached only after a prolonged ramp-up, is consistent with reports that large, multisite pragmatic studies in community practice settings often require extended periods to achieve target sample sizes [21,28]. Seasonal patterns in health care use and regional variation in practice organization contribute to fluctuating recruitment rates, which must be considered in planning and interpreting accrual trajectories [30,31]. Regional heterogeneity in patient demographics, insurance coverage, and practice models influences both participation rates and case mix, potentially affecting complication rates and treatment patterns [7,10]. Documentation of these temporal and regional enrollment patterns will enable sensitivity analysis in the future when estimating peri-implant disease burden and implant survival [7,11,12].

Limitations of this study include a few methodological constraints. The use of convenience sampling based on the willingness of practitioners and patients to participate limits the ability to generalize findings to the broader population [7,10]. Heterogeneity across multiple practitioners and multiple practice environments introduces interoperator variability in diagnostic and treatment approaches, as well as in outcome assessment procedures [14]. While this variability enhances external validity by reflecting real-world practice patterns, it may complicate causal inference and necessitates sensitivity analyses to explore the impact of practice-level effects [14]. Systematic reviews stress that assessing technical and biological events requires a mean observation period of at least 5 years [2,15], whereas monitoring implant loss in high-risk patients often demands follow-up of 10 years or more [4]. Additionally, loss to follow-up is common in prospective registries and may disproportionately affect patients with poorer adherence or complex needs, potentially biasing outcome estimates [1,13] Within a 5- to 7-year funding cycle, the combined demands of start-up, recruitment, and long-term follow-up may not allow for sufficient time to fully characterize late implant failures, particularly among high-risk groups.

Despite these limitations, the registry provides a substantially larger, more diverse, and methodologically rigorous dataset than most previous studies on dental implant outcomes. A key strength of the registry is the combination of high data completeness [22] with broad representation of general practice settings, providing a unique platform for refining contemporary estimates of peri-implant mucositis and peri-implantitis prevalence in the United States [8,14,21]. These data can be directly compared with recent global estimates reporting high patient-level prevalence of peri-implant inflammation and disease, thereby contextualizing US practice-based outcomes within the international literature [7,11,12]. The detailed capture of patient-, implant-, and procedure-level variables enables rigorous evaluation of established and emerging risk indicators, including history of periodontitis, smoking, diabetes, and other systemic or behavioral factors identified as influential in recent meta-analyses [4,11]. Such analyses will be particularly informative for clarifying potential interactions with types of implant fixtures and restoration techniques, which remain incompletely characterized in existing cohort studies [4,10]. Recent evaluations of dental implant prediction models point to limited methodological transparency and poor external validation [14]. By supporting both model development and true external validation in an independent, heterogeneous, practice-based cohort, the Dental Implant Restoration Registry study can contribute to the evolution of more robust clinical prediction tools that support precision dentistry and individualized risk-based prevention strategies [14].

The results of this study have the potential to significantly impact clinical practice by informing evidence-based recommendations for implant therapy, risk assessment, and complication prevention. The establishment of a national implant registry creates opportunities for future targeted studies on specific complications identified through this research. This protocol outlines a comprehensive approach to addressing current knowledge gaps in dental implant and prosthetic outcomes in dental practice settings, contributing to improved patient care and clinical decision-making in implant dentistry.

## Abbreviations

ARC: administrative and resource center
CRF: case report form
IRB: institutional review board
NIDCR: National Institute of Dental and Craniofacial Research
NIH: National Institutes of Health
OHRQoL: oral health–related quality of life
PBRN: Practice-Based Research Network
SPIRIT: Standard Protocol Items: Recommendations for Interventional Trials
STROBE: Strengthening the Reporting of Observational Studies in Epidemiology
